# *cis*-Dichloridobis(triphenyl­phosphine-κ*P*)platinum(II) chloro­form solvate

**DOI:** 10.1107/S1600536809029961

**Published:** 2009-08-08

**Authors:** Jinling Miao, Chunhua Hu, Xiao Feng, Hongwei Chen, Yong Nie

**Affiliations:** a School of Chemistry and Chemical Engineering, University of Jinan, Jinan 250022, People’s Republic of China; b Department of Chemistry, New York University, 100 Washington Square East, New York, NY 10003-6688, USA

## Abstract

In the title compound, [PtCl_2_(C_18_H_15_P)_2_]·CHCl_3_, each Pt^II^ centre adopts a nearly square-planar coordination geometry formed by two P atoms [Pt—P = 2.2481 (17) and 2.2658 (19) Å] and two Cl anions [Pt—Cl = 2.3244 (19) and 2.3548 (17) Å]. The Cl atoms of the chloro­form solvent mol­ecule are disordered over two orientations in a 0.778 (11):0.222 (11) ratio. The crystal packing is stabilized by weak inter­molecular C—H⋯Cl hydrogen bonds, exhib­iting voids with a volume of 215 Å^3^.

## Related literature

For the preparation of *cis*-[PtCl_2_(PPh_3_)_2_], see: Bailar & Itatani (1965 ▶). For the structure of *trans*-[PtCl_2_(PPh_3_)_2_], see: Johansson & Otto (2000 ▶). For the structures of related *cis*-complexes, see: Anderson *et al.* (1982 ▶); Al-Fawaz *et al.* (2004 ▶); Fun *et al.* (2006 ▶).
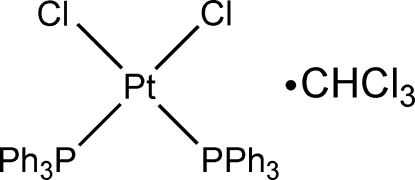


## Experimental

### 

#### Crystal data

[PtCl_2_(C_18_H_15_P)_2_]·CHCl_3_
*M* *_r_* = 909.90
Monoclinic, 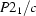

*a* = 10.3174 (9) Å
*b* = 24.436 (2) Å
*c* = 15.6298 (18) Å
β = 98.199 (1)°
*V* = 3900.3 (7) Å^3^
*Z* = 4
Mo *K*α radiation
μ = 4.05 mm^−1^
*T* = 298 K
0.38 × 0.35 × 0.18 mm

#### Data collection

Bruker SMART CCD area-detector diffractometer
Absorption correction: multi-scan (*SADABS*; Bruker, 2001 ▶) *T*_min_ = 0.309, *T*_max_ = 0.530 (expected range = 0.281–0.483)
18772 measured reflections
6877 independent reflections
4984 reflections with *I* > 2σ(*I*)
*R*_int_ = 0.071

#### Refinement

*R*[*F* ^2^ > 2σ(*F* ^2^)] = 0.048
*wR*(*F* ^2^) = 0.119
*S* = 0.97
6877 reflections
434 parameters
H-atom parameters constrained
Δρ_max_ = 1.89 e Å^−3^
Δρ_min_ = −1.27 e Å^−3^

### 

Data collection: *SMART* (Bruker, 2001 ▶); cell refinement: *SAINT* (Bruker, 2001 ▶); data reduction: *SAINT*; program(s) used to solve structure: *SHELXS97* (Sheldrick, 2008 ▶); program(s) used to refine structure: *SHELXL97* (Sheldrick, 2008 ▶); molecular graphics: *SHELXTL* (Sheldrick, 2008 ▶); software used to prepare material for publication: *SHELXTL*.

## Supplementary Material

Crystal structure: contains datablocks I, global. DOI: 10.1107/S1600536809029961/cv2593sup1.cif

Structure factors: contains datablocks I. DOI: 10.1107/S1600536809029961/cv2593Isup2.hkl

Additional supplementary materials:  crystallographic information; 3D view; checkCIF report

## Figures and Tables

**Table 1:** Hydrogen-bond geometry (Å, °)

*D*—H⋯*A*	*D*—H	H⋯*A*	*D*⋯*A*	*D*—H⋯*A*
C3—H3⋯Cl1^i^	0.93	2.80	3.670 (10)	157
C37—H37⋯Cl2^ii^	0.98	2.43	3.390 (15)	165

Symmetry codes: (i) 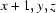; (ii) 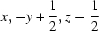.

**Fig. 1.**
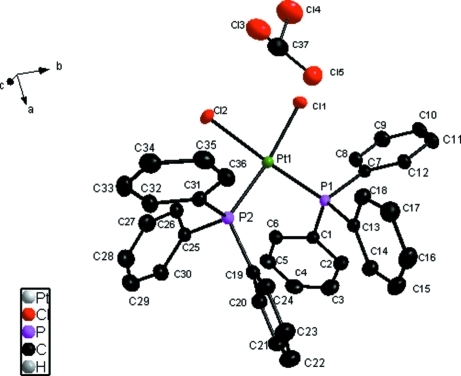
View of (I) with atom labels and 25% probability displacement ellipsoids. For the disordered chloroform molecule, only major part is shown for clarity.

## supplementary crystallographic information

### Comment

*cis*- and *trans*-Dichloridobis (triphenylphosphine)platinum(II) are useful to prepare other platinum complexes. The structures of the *cis*-isomer, either in unsolvated form (Fun *et al.*, 2006) or as an acetone (Anderson *et al.*, 1982) or trichoroform solvate (Al-Fawaz *et al.*, 2004) have been reported. During the course of our studies on platinaborane cluster compounds using the *cis*-isomer as a starting material, we unexpectedly obtained the yellowish crystals of the title compound (I).

As shown in Fig.1, the platinum centre is four-coordinated and adopts a nearly square planar geometry. The Pt—P bond lengths (2.2481 (17) and 2.2658 (19) Å) and Pt—Cl bond lengths (2.3244 (19) and 2.3548 (17) Å), as well as the bond angles around the Pt atom are similar to those in the above-mentioned structures.

Intermolecular C—H···Cl hydrogen bonding is observed between the chloroform and the chloride (Table 1). Moreover, similar C—H···Cl interaction between one of the phenyl groups and the chloride (Table 1) links the molecules into a one-dimensional chain structure.

### Experimental

A mixture of PtCl_2_(PPh_3_)_2_ (314 mg, 0.4 mmol) and (Et_4_N)_2_B_10_H_10_ (150 mg, 0.4 mmol) in HOCH_2_CH_2_OH (38 ml) was heated at 100°C (oil) under N_2_ atomosphere for 39 h. The resulting mixture was filtered to get a red filtrate, to which *ca* 400 ml of water was added. The lower layer formed was separated and treated with water. This process was repeated till the lower layer was colourless. The upper water phase combined was extracted with CH_2_Cl_2_. The yellow organic layer was dried to result in a reddish solid. Recrystalization in CHCl_3_/*n*-hexane(1:4, *V*: *V*) afforded a small amount of yellowish crystals of the title compound suitable for X-ray analysis.

### Refinement

All H-atoms were positioned geometrically and refined using a riding model with d(C—H) = 0.93 Å, *U*_iso_=1.2*U*_eq_ (C) for aromatic, and d(C—H) = 0.98 Å, *U*_iso_=1.5*U*_eq_ (C) for CHCl_3_. The highest residual peak [1.89 eÅ^-3^] and deepest hole [-1.27 eÅ^-3^] are situated 1.05 Å and 0.97 Å, respectively, at atom Pt1.

### Crystal data

**Table:**

[PtCl_2_(C_18_H_15_P)_2_]·CHCl_3_	*F*(000) = 1784
*M**_r_* = 909.90	*D*_x_ = 1.550 Mg m^−^^3^
Monoclinic, *P*2_1_/*c*	Mo *K*α radiation, λ = 0.71073 Å
*a* = 10.3174 (9) Å	Cell parameters from 8493 reflections
*b* = 24.436 (2) Å	θ = 2.2–28.2°
*c* = 15.6298 (18) Å	µ = 4.05 mm^−^^1^
β = 98.199 (1)°	*T* = 298 K
*V* = 3900.3 (7) Å^3^	Block, yellowish
*Z* = 4	0.38 × 0.35 × 0.18 mm

### Data collection

**Table:**

Bruker SMART CCD area-detector diffractometer	6877 independent reflections
Radiation source: fine-focus sealed tube	4984 reflections with *I* > 2σ(*I*)
graphite	*R*_int_ = 0.071
φ and ω scans	θ_max_ = 25.0°, θ_min_ = 1.6°
Absorption correction: multi-scan (*SADABS*; Bruker, 2001)	*h* = −12→12
*T*_min_ = 0.309, *T*_max_ = 0.530	*k* = −29→28
18772 measured reflections	*l* = −14→18

### Refinement

**Table:**

Refinement on *F*^2^	Primary atom site location: structure-invariant direct methods
Least-squares matrix: full	Secondary atom site location: difference Fourier map
*R*[*F*^2^ > 2σ(*F*^2^)] = 0.048	Hydrogen site location: inferred from neighbouring sites
*wR*(*F*^2^) = 0.119	H-atom parameters constrained
*S* = 0.97	*w* = 1/[σ^2^(*F*_o_^2^) + (0.0594*P*)^2^] where *P* = (*F*_o_^2^ + 2*F*_c_^2^)/3
6877 reflections	(Δ/σ)_max_ = 0.001
434 parameters	Δρ_max_ = 1.89 e Å^−^^3^
0 restraints	Δρ_min_ = −1.27 e Å^−^^3^

### Fractional atomic coordinates and isotropic or equivalent isotropic displacement parameters (Å^2^)

**Table:**

	*x*	*y*	*z*	*U*_iso_*/*U*_eq_	Occ. (<1)
Pt1	0.59334 (2)	0.349888 (10)	0.548206 (18)	0.03195 (11)	
Cl1	0.45377 (18)	0.39079 (8)	0.43652 (13)	0.0477 (5)	
Cl2	0.41302 (18)	0.29552 (8)	0.57114 (13)	0.0492 (5)	
Cl3	0.2158 (6)	0.3208 (3)	0.2316 (4)	0.142 (3)	0.778 (11)
Cl4	0.1897 (12)	0.3400 (5)	0.0514 (7)	0.165 (4)	0.778 (11)
Cl5	0.4219 (6)	0.3691 (2)	0.1644 (6)	0.141 (3)	0.778 (11)
Cl3'	0.355 (3)	0.3779 (12)	0.078 (2)	0.197 (14)	0.222 (11)
Cl4'	0.348 (3)	0.3554 (9)	0.2339 (17)	0.156 (12)	0.222 (11)
Cl5'	0.147 (4)	0.3153 (17)	0.081 (3)	0.167 (13)	0.222 (11)
P1	0.75776 (17)	0.40376 (7)	0.51837 (12)	0.0316 (4)	
P2	0.70533 (18)	0.30561 (7)	0.66301 (13)	0.0359 (4)	
C1	0.8924 (7)	0.3665 (3)	0.4836 (5)	0.0416 (18)	
C2	1.0068 (7)	0.3918 (4)	0.4670 (6)	0.054 (2)	
H2	1.0160	0.4295	0.4733	0.065*	
C3	1.1060 (9)	0.3615 (4)	0.4413 (7)	0.072 (3)	
H3	1.1836	0.3786	0.4326	0.086*	
C4	1.0925 (9)	0.3063 (4)	0.4282 (6)	0.068 (3)	
H4	1.1614	0.2861	0.4120	0.081*	
C5	0.9789 (8)	0.2810 (4)	0.4387 (6)	0.061 (2)	
H5	0.9677	0.2439	0.4264	0.074*	
C6	0.8797 (8)	0.3107 (3)	0.4677 (5)	0.048 (2)	
H6	0.8030	0.2930	0.4767	0.058*	
C7	0.7173 (7)	0.4505 (3)	0.4270 (5)	0.0400 (18)	
C8	0.7002 (8)	0.4296 (3)	0.3447 (5)	0.051 (2)	
H8	0.7101	0.3922	0.3367	0.061*	
C9	0.6684 (9)	0.4631 (4)	0.2731 (6)	0.065 (3)	
H9	0.6554	0.4486	0.2175	0.078*	
C10	0.6564 (8)	0.5200 (4)	0.2873 (6)	0.061 (2)	
H10	0.6362	0.5433	0.2403	0.073*	
C11	0.6737 (8)	0.5407 (4)	0.3678 (6)	0.058 (2)	
H11	0.6650	0.5782	0.3760	0.070*	
C12	0.7044 (7)	0.5067 (3)	0.4382 (5)	0.048 (2)	
H12	0.7165	0.5214	0.4936	0.058*	
C13	0.8127 (7)	0.4487 (3)	0.6099 (5)	0.0394 (17)	
C14	0.9418 (8)	0.4603 (3)	0.6409 (5)	0.053 (2)	
H14	1.0093	0.4437	0.6168	0.064*	
C15	0.9694 (10)	0.4972 (4)	0.7088 (6)	0.067 (3)	
H15	1.0560	0.5044	0.7317	0.080*	
C16	0.8705 (10)	0.5230 (4)	0.7418 (7)	0.070 (3)	
H16	0.8902	0.5487	0.7857	0.084*	
C17	0.7427 (10)	0.5116 (3)	0.7111 (6)	0.065 (3)	
H17	0.6756	0.5288	0.7347	0.078*	
C18	0.7138 (8)	0.4744 (3)	0.6452 (5)	0.050 (2)	
H18	0.6268	0.4666	0.6242	0.061*	
C19	0.8622 (7)	0.3305 (3)	0.7180 (5)	0.046 (2)	
C20	0.9782 (8)	0.3178 (3)	0.6859 (6)	0.054 (2)	
H20	0.9755	0.2978	0.6351	0.065*	
C21	1.0977 (9)	0.3351 (4)	0.7302 (7)	0.066 (3)	
H21	1.1753	0.3261	0.7097	0.079*	
C22	1.1006 (10)	0.3649 (4)	0.8029 (8)	0.074 (3)	
H22	1.1808	0.3771	0.8312	0.088*	
C23	0.9906 (10)	0.3775 (4)	0.8358 (7)	0.072 (3)	
H23	0.9962	0.3974	0.8870	0.086*	
C24	0.8702 (9)	0.3613 (3)	0.7945 (6)	0.056 (2)	
H24	0.7945	0.3706	0.8170	0.067*	
C25	0.7459 (7)	0.2350 (3)	0.6415 (5)	0.0449 (19)	
C26	0.6929 (8)	0.2102 (3)	0.5647 (6)	0.053 (2)	
H26	0.6354	0.2294	0.5242	0.063*	
C27	0.7260 (10)	0.1560 (4)	0.5481 (7)	0.072 (3)	
H27	0.6905	0.1390	0.4969	0.086*	
C28	0.8114 (10)	0.1285 (4)	0.6082 (8)	0.075 (3)	
H28	0.8359	0.0929	0.5965	0.090*	
C29	0.8601 (10)	0.1517 (4)	0.6835 (8)	0.073 (3)	
H29	0.9149	0.1316	0.7243	0.088*	
C30	0.8301 (8)	0.2050 (3)	0.7013 (6)	0.057 (2)	
H30	0.8662	0.2210	0.7533	0.069*	
C31	0.6018 (7)	0.3029 (3)	0.7490 (5)	0.0418 (18)	
C32	0.5768 (7)	0.2541 (3)	0.7894 (5)	0.053 (2)	
H32	0.6147	0.2217	0.7739	0.064*	
C33	0.4960 (8)	0.2537 (4)	0.8523 (5)	0.059 (2)	
H33	0.4781	0.2208	0.8782	0.071*	
C34	0.4418 (8)	0.3009 (4)	0.8770 (6)	0.062 (2)	
H34	0.3872	0.3006	0.9195	0.074*	
C35	0.4694 (9)	0.3492 (4)	0.8379 (6)	0.058 (2)	
H35	0.4350	0.3819	0.8555	0.069*	
C36	0.5457 (8)	0.3501 (3)	0.7742 (6)	0.054 (2)	
H36	0.5603	0.3830	0.7473	0.065*	
C37	0.2974 (13)	0.3240 (6)	0.1404 (9)	0.106 (4)	
H37	0.3347	0.2879	0.1316	0.127*	

### Atomic displacement parameters (Å^2^)

**Table:**

	*U*^11^	*U*^22^	*U*^33^	*U*^12^	*U*^13^	*U*^23^
Pt1	0.02648 (17)	0.03000 (17)	0.03899 (18)	−0.00180 (11)	0.00341 (12)	0.00317 (13)
Cl1	0.0357 (10)	0.0525 (12)	0.0525 (12)	0.0011 (9)	−0.0018 (9)	0.0159 (10)
Cl2	0.0363 (11)	0.0537 (12)	0.0566 (12)	−0.0171 (9)	0.0036 (9)	0.0119 (10)
Cl3	0.124 (5)	0.164 (5)	0.145 (5)	0.039 (4)	0.043 (4)	0.027 (4)
Cl4	0.175 (10)	0.188 (10)	0.120 (6)	0.021 (7)	−0.017 (6)	−0.015 (6)
Cl5	0.102 (4)	0.108 (4)	0.212 (10)	−0.007 (3)	0.013 (5)	0.006 (4)
Cl3'	0.20 (3)	0.20 (3)	0.20 (3)	0.00 (2)	0.04 (3)	−0.01 (2)
Cl4'	0.17 (3)	0.149 (19)	0.15 (2)	0.022 (17)	0.006 (18)	−0.001 (15)
Cl5'	0.15 (2)	0.17 (3)	0.18 (3)	−0.004 (19)	0.01 (2)	0.00 (2)
P1	0.0273 (10)	0.0259 (9)	0.0416 (11)	−0.0011 (7)	0.0046 (8)	0.0021 (8)
P2	0.0331 (11)	0.0335 (10)	0.0403 (11)	−0.0019 (8)	0.0019 (9)	0.0046 (9)
C1	0.034 (4)	0.039 (4)	0.052 (5)	−0.001 (3)	0.007 (4)	0.003 (4)
C2	0.041 (5)	0.059 (5)	0.065 (6)	−0.003 (4)	0.015 (4)	0.000 (5)
C3	0.049 (6)	0.088 (8)	0.083 (7)	−0.004 (5)	0.025 (5)	−0.008 (6)
C4	0.051 (6)	0.080 (7)	0.074 (7)	0.020 (5)	0.016 (5)	−0.003 (6)
C5	0.058 (6)	0.057 (6)	0.069 (6)	0.019 (5)	0.011 (5)	−0.006 (5)
C6	0.045 (5)	0.048 (5)	0.054 (5)	0.004 (4)	0.012 (4)	0.004 (4)
C7	0.035 (4)	0.037 (4)	0.049 (5)	0.000 (3)	0.010 (4)	0.009 (4)
C8	0.055 (5)	0.047 (5)	0.052 (5)	0.002 (4)	0.014 (4)	0.009 (4)
C9	0.071 (7)	0.069 (6)	0.057 (6)	0.002 (5)	0.013 (5)	0.014 (5)
C10	0.064 (6)	0.057 (6)	0.062 (6)	0.012 (4)	0.014 (5)	0.027 (5)
C11	0.062 (6)	0.047 (5)	0.067 (6)	0.008 (4)	0.013 (5)	0.013 (5)
C12	0.049 (5)	0.040 (5)	0.056 (5)	0.002 (4)	0.008 (4)	0.005 (4)
C13	0.036 (4)	0.033 (4)	0.048 (5)	−0.004 (3)	0.003 (4)	0.002 (4)
C14	0.046 (5)	0.052 (5)	0.061 (5)	−0.006 (4)	0.005 (4)	−0.001 (5)
C15	0.061 (6)	0.063 (6)	0.073 (7)	−0.016 (5)	−0.004 (5)	−0.007 (6)
C16	0.080 (8)	0.056 (6)	0.072 (7)	−0.013 (5)	0.000 (6)	−0.013 (5)
C17	0.067 (7)	0.056 (6)	0.072 (6)	0.002 (5)	0.008 (5)	−0.014 (5)
C18	0.044 (5)	0.048 (5)	0.059 (6)	−0.003 (4)	0.006 (4)	−0.004 (4)
C19	0.041 (5)	0.043 (4)	0.053 (5)	0.000 (4)	−0.001 (4)	0.011 (4)
C20	0.049 (6)	0.054 (5)	0.058 (6)	0.000 (4)	0.001 (4)	0.007 (5)
C21	0.043 (6)	0.069 (6)	0.082 (7)	0.001 (5)	−0.001 (5)	0.010 (6)
C22	0.057 (7)	0.080 (7)	0.079 (8)	−0.007 (5)	−0.009 (6)	−0.003 (6)
C23	0.067 (7)	0.072 (7)	0.070 (7)	−0.010 (5)	−0.008 (6)	−0.007 (6)
C24	0.052 (6)	0.058 (5)	0.055 (6)	−0.002 (4)	0.000 (4)	−0.002 (5)
C25	0.042 (5)	0.037 (4)	0.055 (5)	−0.001 (3)	0.004 (4)	0.004 (4)
C26	0.052 (5)	0.043 (5)	0.064 (6)	−0.001 (4)	0.009 (4)	0.004 (5)
C27	0.075 (7)	0.059 (6)	0.079 (7)	−0.002 (5)	0.005 (6)	−0.009 (5)
C28	0.075 (8)	0.053 (6)	0.096 (9)	0.008 (5)	0.010 (7)	−0.001 (6)
C29	0.070 (7)	0.059 (6)	0.087 (8)	0.010 (5)	0.003 (6)	0.012 (6)
C30	0.060 (6)	0.046 (5)	0.065 (6)	0.003 (4)	0.001 (5)	0.007 (5)
C31	0.039 (4)	0.044 (5)	0.042 (5)	−0.005 (3)	0.006 (4)	0.005 (4)
C32	0.057 (6)	0.049 (5)	0.054 (5)	−0.001 (4)	0.008 (4)	0.011 (4)
C33	0.066 (6)	0.060 (6)	0.052 (5)	−0.011 (5)	0.010 (5)	0.019 (5)
C34	0.054 (6)	0.077 (7)	0.056 (6)	0.005 (5)	0.013 (5)	0.012 (5)
C35	0.052 (6)	0.064 (6)	0.058 (6)	0.012 (4)	0.010 (5)	0.004 (5)
C36	0.055 (6)	0.054 (5)	0.055 (6)	0.005 (4)	0.010 (5)	0.014 (4)
C37	0.102 (11)	0.098 (9)	0.115 (11)	0.020 (8)	0.009 (9)	−0.023 (9)

### Geometric parameters (Å, °)

**Table:**

Pt1—P1	2.2481 (17)	C15—C16	1.362 (12)
Pt1—P2	2.2658 (19)	C15—H15	0.9300
Pt1—Cl1	2.3244 (19)	C16—C17	1.367 (12)
Pt1—Cl2	2.3548 (17)	C16—H16	0.9300
Cl3—C37	1.758 (14)	C17—C18	1.373 (11)
Cl4—C37	1.697 (17)	C17—H17	0.9300
Cl5—C37	1.693 (15)	C18—H18	0.9300
Cl3'—C37	1.79 (3)	C19—C20	1.397 (11)
Cl4'—C37	1.67 (3)	C19—C24	1.404 (11)
Cl5'—C37	1.70 (5)	C20—C21	1.390 (12)
P1—C1	1.809 (7)	C20—H20	0.9300
P1—C13	1.828 (8)	C21—C22	1.347 (14)
P1—C7	1.829 (7)	C21—H21	0.9300
P2—C25	1.818 (7)	C22—C23	1.347 (13)
P2—C19	1.825 (8)	C22—H22	0.9300
P2—C31	1.833 (7)	C23—C24	1.375 (12)
C1—C6	1.388 (10)	C23—H23	0.9300
C1—C2	1.389 (10)	C24—H24	0.9300
C2—C3	1.369 (11)	C25—C26	1.387 (11)
C2—H2	0.9300	C25—C30	1.390 (11)
C3—C4	1.368 (12)	C26—C27	1.399 (11)
C3—H3	0.9300	C26—H26	0.9300
C4—C5	1.356 (12)	C27—C28	1.369 (14)
C4—H4	0.9300	C27—H27	0.9300
C5—C6	1.383 (10)	C28—C29	1.338 (14)
C5—H5	0.9300	C28—H28	0.9300
C6—H6	0.9300	C29—C30	1.377 (11)
C7—C8	1.372 (10)	C29—H29	0.9300
C7—C12	1.394 (10)	C30—H30	0.9300
C8—C9	1.386 (11)	C31—C36	1.374 (10)
C8—H8	0.9300	C31—C32	1.390 (10)
C9—C10	1.415 (12)	C32—C33	1.376 (10)
C9—H9	0.9300	C32—H32	0.9300
C10—C11	1.344 (12)	C33—C34	1.362 (11)
C10—H10	0.9300	C33—H33	0.9300
C11—C12	1.379 (10)	C34—C35	1.378 (11)
C11—H11	0.9300	C34—H34	0.9300
C12—H12	0.9300	C35—C36	1.353 (12)
C13—C18	1.380 (10)	C35—H35	0.9300
C13—C14	1.380 (10)	C36—H36	0.9300
C14—C15	1.391 (11)	C37—H37	0.9800
C14—H14	0.9300		
			
P1—Pt1—P2	97.43 (7)	C24—C19—P2	121.3 (6)
P1—Pt1—Cl1	89.85 (7)	C21—C20—C19	119.9 (9)
P2—Pt1—Cl1	172.47 (6)	C21—C20—H20	120.1
P1—Pt1—Cl2	176.28 (7)	C19—C20—H20	120.1
P2—Pt1—Cl2	86.26 (7)	C22—C21—C20	119.7 (9)
Cl1—Pt1—Cl2	86.48 (7)	C22—C21—H21	120.2
C1—P1—C13	111.8 (3)	C20—C21—H21	120.2
C1—P1—C7	100.3 (3)	C23—C22—C21	121.9 (10)
C13—P1—C7	104.3 (3)	C23—C22—H22	119.1
C1—P1—Pt1	113.7 (2)	C21—C22—H22	119.1
C13—P1—Pt1	110.4 (2)	C22—C23—C24	120.5 (10)
C7—P1—Pt1	115.6 (2)	C22—C23—H23	119.7
C25—P2—C19	101.0 (4)	C24—C23—H23	119.7
C25—P2—C31	105.9 (3)	C23—C24—C19	119.6 (9)
C19—P2—C31	103.7 (4)	C23—C24—H24	120.2
C25—P2—Pt1	114.3 (3)	C19—C24—H24	120.2
C19—P2—Pt1	122.1 (3)	C26—C25—C30	118.9 (7)
C31—P2—Pt1	108.3 (3)	C26—C25—P2	120.2 (6)
C6—C1—C2	117.7 (7)	C30—C25—P2	121.0 (6)
C6—C1—P1	119.4 (5)	C25—C26—C27	119.8 (9)
C2—C1—P1	122.8 (6)	C25—C26—H26	120.1
C3—C2—C1	120.3 (8)	C27—C26—H26	120.1
C3—C2—H2	119.9	C28—C27—C26	119.2 (10)
C1—C2—H2	119.9	C28—C27—H27	120.4
C4—C3—C2	120.8 (9)	C26—C27—H27	120.4
C4—C3—H3	119.6	C29—C28—C27	121.3 (10)
C2—C3—H3	119.6	C29—C28—H28	119.4
C5—C4—C3	120.2 (8)	C27—C28—H28	119.4
C5—C4—H4	119.9	C28—C29—C30	120.8 (10)
C3—C4—H4	119.9	C28—C29—H29	119.6
C4—C5—C6	119.5 (9)	C30—C29—H29	119.6
C4—C5—H5	120.2	C29—C30—C25	120.0 (9)
C6—C5—H5	120.2	C29—C30—H30	120.0
C5—C6—C1	121.2 (8)	C25—C30—H30	120.0
C5—C6—H6	119.4	C36—C31—C32	118.4 (7)
C1—C6—H6	119.4	C36—C31—P2	119.6 (6)
C8—C7—C12	118.8 (7)	C32—C31—P2	122.0 (6)
C8—C7—P1	119.0 (6)	C33—C32—C31	120.1 (8)
C12—C7—P1	122.2 (6)	C33—C32—H32	119.9
C7—C8—C9	121.4 (8)	C31—C32—H32	119.9
C7—C8—H8	119.3	C34—C33—C32	120.7 (8)
C9—C8—H8	119.3	C34—C33—H33	119.6
C8—C9—C10	118.0 (9)	C32—C33—H33	119.6
C8—C9—H9	121.0	C33—C34—C35	118.8 (8)
C10—C9—H9	121.0	C33—C34—H34	120.6
C11—C10—C9	120.9 (8)	C35—C34—H34	120.6
C11—C10—H10	119.5	C36—C35—C34	121.1 (8)
C9—C10—H10	119.5	C36—C35—H35	119.4
C10—C11—C12	120.2 (8)	C34—C35—H35	119.4
C10—C11—H11	119.9	C35—C36—C31	120.8 (8)
C12—C11—H11	119.9	C35—C36—H36	119.6
C11—C12—C7	120.6 (8)	C31—C36—H36	119.6
C11—C12—H12	119.7	Cl4'—C37—Cl5	51.1 (10)
C7—C12—H12	119.7	Cl4'—C37—Cl4	134.5 (11)
C18—C13—C14	119.9 (7)	Cl5—C37—Cl4	114.9 (10)
C18—C13—P1	114.9 (6)	Cl4'—C37—Cl5'	133 (2)
C14—C13—P1	125.1 (6)	Cl5—C37—Cl5'	143.7 (16)
C13—C14—C15	118.9 (8)	Cl4—C37—Cl5'	31.2 (11)
C13—C14—H14	120.5	Cl4'—C37—Cl3	55.8 (11)
C15—C14—H14	120.5	Cl5—C37—Cl3	106.5 (8)
C16—C15—C14	120.4 (9)	Cl4—C37—Cl3	110.0 (9)
C16—C15—H15	119.8	Cl5'—C37—Cl3	86.0 (16)
C14—C15—H15	119.8	Cl4'—C37—Cl3'	92.8 (15)
C15—C16—C17	120.7 (9)	Cl5—C37—Cl3'	48.8 (12)
C15—C16—H16	119.7	Cl4—C37—Cl3'	67.4 (13)
C17—C16—H16	119.7	Cl5'—C37—Cl3'	98.4 (17)
C16—C17—C18	119.6 (9)	Cl3—C37—Cl3'	135.1 (12)
C16—C17—H17	120.2	Cl4'—C37—H37	117.1
C18—C17—H17	120.2	Cl5—C37—H37	108.4
C17—C18—C13	120.5 (8)	Cl4—C37—H37	108.4
C17—C18—H18	119.8	Cl5'—C37—H37	99.0
C13—C18—H18	119.8	Cl3—C37—H37	108.4
C20—C19—C24	118.4 (8)	Cl3'—C37—H37	114.8
C20—C19—P2	120.3 (7)		
			
P2—Pt1—P1—C1	65.2 (3)	Pt1—P1—C13—C14	137.5 (6)
Cl1—Pt1—P1—C1	−116.7 (3)	C18—C13—C14—C15	1.2 (12)
Cl2—Pt1—P1—C1	−107.5 (11)	P1—C13—C14—C15	177.1 (6)
P2—Pt1—P1—C13	−61.5 (3)	C13—C14—C15—C16	−2.4 (13)
Cl1—Pt1—P1—C13	116.6 (3)	C14—C15—C16—C17	2.4 (14)
Cl2—Pt1—P1—C13	125.9 (10)	C15—C16—C17—C18	−1.2 (14)
P2—Pt1—P1—C7	−179.5 (3)	C16—C17—C18—C13	0.0 (13)
Cl1—Pt1—P1—C7	−1.4 (3)	C14—C13—C18—C17	0.0 (12)
Cl2—Pt1—P1—C7	7.8 (11)	P1—C13—C18—C17	−176.4 (6)
P1—Pt1—P2—C25	−107.0 (3)	C25—P2—C19—C20	44.4 (7)
Cl1—Pt1—P2—C25	87.8 (6)	C31—P2—C19—C20	153.9 (6)
Cl2—Pt1—P2—C25	72.6 (3)	Pt1—P2—C19—C20	−83.8 (7)
P1—Pt1—P2—C19	15.1 (3)	C25—P2—C19—C24	−133.6 (7)
Cl1—Pt1—P2—C19	−150.1 (6)	C31—P2—C19—C24	−24.0 (7)
Cl2—Pt1—P2—C19	−165.4 (3)	Pt1—P2—C19—C24	98.3 (7)
P1—Pt1—P2—C31	135.3 (3)	C24—C19—C20—C21	0.6 (12)
Cl1—Pt1—P2—C31	−30.0 (6)	P2—C19—C20—C21	−177.4 (6)
Cl2—Pt1—P2—C31	−45.2 (3)	C19—C20—C21—C22	−1.3 (13)
C13—P1—C1—C6	133.7 (6)	C20—C21—C22—C23	1.8 (16)
C7—P1—C1—C6	−116.3 (7)	C21—C22—C23—C24	−1.6 (17)
Pt1—P1—C1—C6	7.8 (7)	C22—C23—C24—C19	1.0 (14)
C13—P1—C1—C2	−50.2 (8)	C20—C19—C24—C23	−0.5 (12)
C7—P1—C1—C2	59.9 (8)	P2—C19—C24—C23	177.5 (7)
Pt1—P1—C1—C2	−176.1 (6)	C19—P2—C25—C26	−142.3 (6)
C6—C1—C2—C3	−4.2 (13)	C31—P2—C25—C26	109.8 (7)
P1—C1—C2—C3	179.6 (7)	Pt1—P2—C25—C26	−9.3 (7)
C1—C2—C3—C4	2.6 (15)	C19—P2—C25—C30	37.7 (7)
C2—C3—C4—C5	1.5 (16)	C31—P2—C25—C30	−70.2 (7)
C3—C4—C5—C6	−3.8 (15)	Pt1—P2—C25—C30	170.7 (6)
C4—C5—C6—C1	2.2 (13)	C30—C25—C26—C27	−0.8 (12)
C2—C1—C6—C5	1.8 (12)	P2—C25—C26—C27	179.1 (6)
P1—C1—C6—C5	178.2 (7)	C25—C26—C27—C28	−0.4 (14)
C1—P1—C7—C8	49.8 (7)	C26—C27—C28—C29	2.3 (16)
C13—P1—C7—C8	165.7 (6)	C27—C28—C29—C30	−2.8 (17)
Pt1—P1—C7—C8	−72.9 (6)	C28—C29—C30—C25	1.5 (15)
C1—P1—C7—C12	−129.5 (6)	C26—C25—C30—C29	0.3 (12)
C13—P1—C7—C12	−13.7 (7)	P2—C25—C30—C29	−179.6 (7)
Pt1—P1—C7—C12	107.8 (6)	C25—P2—C31—C36	−174.3 (7)
C12—C7—C8—C9	−1.1 (12)	C19—P2—C31—C36	79.7 (7)
P1—C7—C8—C9	179.5 (6)	Pt1—P2—C31—C36	−51.3 (7)
C7—C8—C9—C10	1.2 (13)	C25—P2—C31—C32	5.0 (8)
C8—C9—C10—C11	−0.8 (13)	C19—P2—C31—C32	−100.9 (7)
C9—C10—C11—C12	0.3 (13)	Pt1—P2—C31—C32	128.1 (6)
C10—C11—C12—C7	−0.2 (12)	C36—C31—C32—C33	0.9 (12)
C8—C7—C12—C11	0.6 (11)	P2—C31—C32—C33	−178.5 (6)
P1—C7—C12—C11	180.0 (6)	C31—C32—C33—C34	−1.4 (13)
C1—P1—C13—C18	−174.0 (6)	C32—C33—C34—C35	0.0 (13)
C7—P1—C13—C18	78.5 (6)	C33—C34—C35—C36	1.9 (14)
Pt1—P1—C13—C18	−46.3 (6)	C34—C35—C36—C31	−2.4 (14)
C1—P1—C13—C14	9.9 (8)	C32—C31—C36—C35	1.0 (13)
C7—P1—C13—C14	−97.7 (7)	P2—C31—C36—C35	−179.7 (7)

### Hydrogen-bond geometry (Å, °)

**Table:**

*D*—H···*A*	*D*—H	H···*A*	*D*···*A*	*D*—H···*A*
C3—H3···Cl1^i^	0.93	2.80	3.670 (10)	157
C37—H37···Cl2^ii^	0.98	2.43	3.390 (15)	165

Symmetry codes: (i) *x*+1, *y*, *z*; (ii) *x*, −*y*+1/2, *z*−1/2.
